# Health Effects Associated with Exposures to Endocrine Disrupting Chemicals

**DOI:** 10.3390/toxics10080425

**Published:** 2022-07-28

**Authors:** Gunnar Toft, Zeyan Liew

**Affiliations:** 1 Steno Diabetes Center Aarhus, Aarhus University Hospital, 8200 Aarhus, Denmark; 2 Department of Public Health, Aarhus University, Bartholins Allé 2—Building 1260, 8000 Aarhus, Denmark; 3 Department of Environmental Health Sciences, Yale School of Public Health, 60 College Street, New Haven, CT 06510, USA

A number of recent studies have shown that human exposures to a wide range of endocrine-disrupting chemicals (EDCs) may increase the risk of disease across the lifespan by altering the homeostasis or action of endogenous hormones, or other signaling chemicals of the endocrine system. Commonly investigated EDCs include chemicals widely applied in commercial and industrial products, such as per- and poly-fluoralkyl substances (PFAS), polychlorinated biphenyls (PCBs), bisphenol A (BPA), phthalates, and certain pesticides. Our understanding of human health risks associated with exposure to EDCs remains limited. The early development period (i.e., in-utero, infancy, and childhood) might be more vulnerable to exposures to EDCs, but depending on the outcomes, relevant exposure windows may include several periods throughout the entire lifespan. In this Special Issue, we include seven epidemiological studies that address different aspects of human health risks associated with exposures to EDCs.

Two epidemiological studies assessed potential associations between EDCs exposure and human reproductive health effects. First, an Italian study considered serum and follicular level of phthalates and BPA among 122 women undergoing oocyte retrieval for in vitro fertilization [1]. They found widespread exposure to phthalates with detection of monobutyl phthalate (MBP) in >99% of follicular fluid and serum samples and monoethylhexyl phthalate (MEHP) in 80% of follicular fluid samples and 95% of serum samples. BPA was detected in 29% of follicular fluid samples and 52% of serum samples. The study reported that geographical area of the study participants, their body mass index at the time of study, and the utilization of plastic food packaging were significantly associated with the BPA and phthalates exposure levels. However, eating habits reported by the participants were not associated with exposure to these EDCs. A major finding of the study was that women with detected higher follicular fluid MBP levels experienced more irregular menstrual cycles. The findings of the study suggest future research should look into the adverse health effects of MBP, especially measured in the follicular fluid, on female fertility and also fetal development.

Next, a cross sectional study of 1058 Danish men (age 18–21) evaluated whether the use of personal care products (PCPs) that potentially contain multiple EDCs affected semen quality [2]. Semi-quantitative measures of exposure to PCPs were generated using at least weekly self-reported use of the 12 most common categories of the PCPs. Semen quality of the Danish men was evaluated according to volume, motility, total sperm count, sperm concentration, and morphology. Overall, the study did not find consistent associations between the self-reported PCP use and each of the semen quality measures. However, this study did not directly examine a specific type or class of EDC and future research into EDCs exposure effect on human semen quality is still warranted.

The following two studies published in this issue evaluated the effect of EDCs on maternal health, including preeclampsia and adiposity. The risk of developing preeclampsia in pregnancy was evaluated in relation to exposure to PFAS in a case-control study from Sweden. The study includes 296 cases diagnosed with preeclampsia and 580 controls pregnancies without preeclampsia identified through the Swedish medical birth register [3]. PFAS was analyzed using maternal serum samples from all included women collected in early pregnancy before the preeclampsia diagnosis. The samples were analyzed for the specific PFAS: perfluorooctanoic acid (PFOA), perfluorooctane sulfonate (PFOS), perfluorononanoic acid (PFNA), and perfluorohexane sulfonate (PFHxS) using liquid chromatography-tandem-mass-spectrometry (LC/MS/MS). The study found slightly increased odds of preeclampsia at higher tertiles of PFNA and PFHxS, but most effect estimates were not statistically significant. The study concluded there is limited support for an association between prenatal PFAS exposure and the risk of preeclampsia in Sweden.

A study in a population with a high prevalence of obesity from the Pacific Island nation of Samoa evaluated the association of dietary BPA exposure and adiposity among 399 mother–child pairs [4]. The study estimated the maternal and children dietary BPA exposure index semi-quantitatively based on the sum of self-reported dietary intake of food items that are likely to contain BPA. The study reported that socioeconomic status was a strong determinant of the estimated BPA exposure in both the mothers and the children. However, the study found no significant associations between the estimated dietary BPA index in mothers or children and their respective body mass index or abdominal circumference. The high background level of obesity in this population with an average body mass index of 34.9 may have masked any BPA-related associations on adiposity. Moreover, omitting BPA exposure from unknown dietary or non-dietary sources in this population may induce measurement errors of exposure and possibly bias the results towards the null.

Next, we have also included two articles that assessed associations between EDC chemical exposures and neurological outcomes in children. A California study evaluated whether maternal pregnancy exposure to agricultural pesticide compounds affects offspring’s risk for cerebral palsy (CP) [5]. CP is the most common neuromotor disorder in childhood with largely unknown etiology for the majority of the cases and few perinatal risk factors have been identified. The study linked maternal residential addresses at birth to the California Pesticide Use Reporting database to assess maternal residential exposures to frequently applied pesticides with documented endocrine-disrupting actions. The study reported maternal first trimester exposures to 15 pesticides suspected to affect the estrogen, and 7 pesticides suspected to affect the thyroid hormone system, were associated with a modest increase in risk for CP in the female offspring. Findings of this study that reported pesticide exposures as a potential environmental risk factor for CP are novel and would likely inspire additional research into the roles of EDCs in the development of CP and other neuromotor diseases.

Another study from Taiwan examined childhood urinary levels of multiple EDCs, including several phthalates metabolites, para-hydroxybenzoic acids, and BPA, on the susceptibility to attention-deficit/hyperactivity disorder (ADHD) [6]. ADHD is the most common neurobehavioral disorder in childhood and is four times more common in boys than girls. The study also assessed whether the EDC exposure was associated with child serum gonadal hormones, including follicle-stimulating hormone (FSH), luteinizing hormone (LH), testosterone, free testosterone, estradiol, progesterone, sex hormone-binding globulin (SHBG), and prolactin. The study reported urine MBzP and MEHP levels were positively correlated with serum testosterone levels among 98 boys affected by ADHD, and in 32 girls with ADHD urine phthalate (MEP) level was associated with serum LH, testosterone, and free testosterone levels. The study comparison groups include 42 boys and 26 girls without ADHD, and any other psychiatric disorders, respectively. The study concluded that EDCs exposure may affect gonadal hormones in boys and girls and hence the susceptibility for ADHD. Longitudinal data with a larger sample size would be needed to confirm these innovative research findings.

Finally, this issue also included a methodological study that explored the challenges when distinguishing the confounding or mediating role of obesity in epidemiological analyses that aim to estimate EDCs exposure effect on other chronic health outcomes [7]. The study used PFAS exposure analyses as examples. The article developed directed acyclic graphs to describe a collider bias that could theoretically be introduced when adjusting or stratifying body weight measure as an intermediate variable in PFAS exposure analyses. The study further showed that heterogeneity of results is observed when stratifying participants’ status as overweight or obese in two cross-sectional studies of PFAS and thyroid function or cardiovascular disease risk associations. However, because of the lack of clear time-ordering and latency of PFAS and body weight measures in these cross-sectional data, these stratified analyses do not distinguish whether the heterogeneity results by obesity status are due to confounding, effect modifications, or collider–stratification bias. This study shows that a seemingly simple stratification analysis by body weight in PFAS analyses can possibly yield results that are difficult to interpret. Careful consideration of the confounding or mediating role of body weight measures in EDCs health research is warranted.

In summary, the seven manuscripts included in the present Special Issue highlight the complexity when studying human health effects associated with endocrine-disrupting chemicals. The included papers demonstrated small steps in our continued understanding of the exposure effects of various EDCs on human health. This area of research is far from complete and greater efforts would be needed from the scientific community to understand whether and how these extremely widespread chemicals may influence health and disease risk in the populations.
